# A Scoping Literature Review of Pancreatic Panniculitis

**DOI:** 10.7759/cureus.78000

**Published:** 2025-01-26

**Authors:** Erin R Pomerantz, Madison A Hackley, Caitlin E Lawlor, Diane Lee, Daphne Thampy, Nicholas D Brownstone, Kiran Motaparthi, Sylvia Hsu

**Affiliations:** 1 Department of Dermatology, Lewis Katz School of Medicine at Temple University, Philadelphia, USA; 2 Department of Dermatology, University of Florida, Gainesville, USA

**Keywords:** pancreatic acinar cell carcinoma, pancreatic panniculitis, pancreatitis, panniculitis, subcutaneous nodules

## Abstract

Pancreatic panniculitis (PP) is a rare cutaneous manifestation of pancreatic disease, often presenting as tender, erythematous, subcutaneous nodules, typically on the lower extremities. It can also affect the upper extremities, buttocks, and abdomen. PP is associated with conditions such as acute pancreatitis, pancreatic acinar cell carcinoma, and pancreatic adenocarcinoma. In some cases, PP occurs as part of pancreatitis, panniculitis, and polyarthritis (PPP) syndrome, a clinical trial that also involves aseptic polyarthritis. While the pathophysiology of PP is not fully understood, cutaneous findings often resolve as pancreatic enzyme levels normalize. This study aims to summarize and characterize the demographics, distribution, morphology, symptoms, etiology, management, and outcomes of PP.

## Introduction and background

Pancreatic panniculitis (PP) has an estimated prevalence ranging from 0.3% to 3% in patients with pancreatic disease [1]. Due to its rarity, significant gaps remain in understanding the presentation, etiology, and management of the disease. PP typically affects middle-aged men, particularly those with chronic alcoholism and pancreatitis. While pancreatic acinar cell carcinoma (ACC) is rare, accounting for only 1% of pancreatic cancers, approximately 16% of patients with ACC develop PP [2]. PP can occur at any stage of underlying pancreatic disease, and cutaneous findings are often the primary reason for initial presentation to healthcare providers, as observed in 40% of cases [3].

Diagnosis of PP typically involves elevated lipase and amylase levels, with lipase causing fat necrosis in subcutaneous tissue. Histologically, PP is characterized by neutrophilic lobular panniculitis with fat necrosis and “ghost cells” (anucleate adipocytes) [4,5]. Elevated amylase and lipase levels are also used to help diagnose acute pancreatitis, but they may also be elevated in other pancreatic diseases. The management of PP is largely determined by the underlying pancreatic disease. In cases where PP is secondary to acute pancreatitis, supportive care including intravenous hydration, bowel rest, and non-steroidal anti-inflammatory drugs (NSAIDs), is commonly used. In these patients, cutaneous findings generally resolve once the pancreatic symptoms improve [6]. However, PP secondary to pancreatic tumors is more challenging to treat and often requires a combination of surgery, chemotherapy, and supportive care. Given the rarity of PP and varied clinical presentations, we conducted this review to enhance understanding, improve diagnostic accuracy, and optimize patient outcomes.

## Review

Objective

This scoping literature review was conducted to discover trends in the underlying etiology, timing of skin lesions, onset of pancreatic disease, morphology, pancreatic enzyme levels, management, and outcome of patients diagnosed with PP given the rarity of its presentation.

Research methodology

Case reports were sourced from PubMed (Medline), Embase, CINAHL, Web of Science, and Grey literature resources: Clinicaltrials.gov, and Google Scholar. Search terms included “pancreatic panniculitis,” “pancreatitis AND rash OR panniculitis,” “pancreatic carcinoma AND rash OR panniculitis,” “pancreatic pseudocyst AND rash OR panniculitis,” “pancreas divisum AND rash OR panniculitis,” “traumatic pancreatitis AND rash OR panniculitis,” and the mesh terms ("pancreatic diseases"[Mesh]) AND "panniculitis"[Mesh]).

Inclusion criteria were English-language case reports describing underlying pancreatic disease, the morphology and onset of skin lesions, management, and outcomes. Exclusion criteria included duplicate articles, reviews/meta-analyses, and cases lacking information on the underlying disease or cutaneous manifestations. Throughout the data extraction phase, 99 additional case reports were excluded because they did not satisfy inclusion/exclusion criteria, were no longer available online, or were duplicate articles. Ultimately, 163 cases of PP were included. Descriptive statistics were used to analyze the data, with percentages applied across all categories and mean values specifically applied to age and sex variables.

Results

Patient Demographics

The majority of patients with PP were male (62.5%), with an average age of 57 years (Table 1). The average lipase and amylase levels were 12,306 U/L and 3,008 U/L, respectively (Table 1).

**Table 1:** Demographics of Patients With PP PP: Pancreatic panniculitis

Percentage of Male Patients	Percentage of Female Patients	Average Age of Patients	Average Lipase (U/L)	Average Amylase (U/L)
62.5	37.5	57 years	12,306	3,008

Etiology

Acute pancreatitis was the most common underlying condition, accounting for 28.8% of cases (Table 2). Pancreatitis secondary to other diseases and pancreatic ACC followed as the second and third most common causes, respectively (Table 2).

**Table 2:** Underlying Pancreatic Diseases, Ulceration in PP, and Outcomes PP: Pancreatic panniculitis; ERCP: Endoscopic retrograde cholangiopancreatography

Etiology	Number of Total Cases (%)	Number of Cases With No Ulceration (%)	Number of Cases With >1 Ulceration (%)	Number of Cases With Complete Resolution of PP (%)	Number of Cases Resulting in Death (%)	Number of Cases With Partial Resolution of PP (%)
Pancreatic Adenocarcinoma	10 (6.13)	10 (100)	0 (0.0)	5 (50)	2 (20)	1 (100)
Pancreatic Acinar Cell Carcinoma	27 (16.56)	18 (66.7)	5 (18.5)	10 (37.0)	12 (44.4)	3 (11.1)
Neuroendocrine Tumor of Pancreas	6 (3.68)	3 (50)	2 (33)	0 (0.0)	5 (83.3)	0 (0.0)
Pancreatic Carcinoma, Unspecified/Other	14 (8.59)	11 (78.6)	3 (21.4)	4 (28.6)	9 (64.3)	1 (7.1)
Intrahepatic Cholangiocarcinoma	1 (0.61)	1 (100)	0 (0.0)	1 (100)	0 (0.0)	0 (0.0)
Acute Pancreatitis	47 (28.83)	38 (80.9)	9 (19.1)	35 (74.5)	9 (19.2)	1 (2.1)
Pancreatitis, Unspecified	12 (7.36)	10 (83.3)	1 (8.3)	11 (91.7)	0 (0.0)	1 (9.1)
Chronic Pancreatitis	8 (4.91)	7 (87.5)	1 (12.5)	7 (87.5)	1 (12.5)	0 (0.0)
Pancreatitis Secondary to Other Disease	30 (18.4)	29 (96.7)	1 (3.3)	27 (90.0)	2 (6.7)	0 (0.0)
Pancreatitis from ERCP	2 (1.23)	2 (100)	0 (0.0)	1 (50.0)	0 (0.0)	1 (50.0)
Fistula	3 (1.84)	1 (33.3)	2 (66.7)	2 (66.7)	1 (33.3)	0 (0.0)
Other/Unknown	3 (1.84)	2 (66.7)	1 (33.3)	1 (33.3)	1 (33.3)	1 (33.3)
Total Cases	163	132	25	104	42	9

Morphology

Lesions were consistently described as "erythematous, subcutaneous nodules" across all case reports. The size of the lesions ranged from 5 mm to 5 cm. Pain and tenderness were the most reported symptoms, with 120 cases describing lesions as “painful and/or tender.” Other descriptors included "blue/violaceous/hyperpigmented" lesions in 21 cases, and 22 cases had concurrent arthritis. Additional features included “draining yellow/brown oil exudate,” “ill-defined” borders, and associated “heat/warmth.” A detailed list of morphological descriptions can be found in Table 3.

**Table 3:** Morphology of PP PP: Pancreatic panniculitis

Morphology Description	Number of Lesions (%)	Morphology Description	Number of Lesions
Erythematous Subcutaneous Nodules	163 (100)	Soft	2 (1.2)
Painful/Tender	120 (73.6)	Varying Consistency	1 (0.61)
Pruritic	2 (1.2)	Dusky/Necrotic	4 (2.5)
Blue/Violaceous/Hyperpigmented	21 (12.9)	Fluctuance	8 (5.0)
Plaques Associated With Nodules	10 (6.1)	Heat/Warmth	11 (6.7)
Nodular Panniculitis	1 (0.61)	Purulent	1 (0.61)
Associated Arthritis	22 (13.5)	Cystic	1 (0.61)
Ill-Defined	14 (8.6)	Background Erythema	2 (1.2)
Draining - Yellow/Brown Oily Exudate	15 (9.2)	Rubbery	1 (0.61)
Firm	5 (3.1)	Started Annular Pink Blanching	1 (0.61)
Mobile	1 (0.61)		

Lesion Location

Lesions occurred on the lower extremities in 152 of the cases (Table 4). A total of 135 cases were described as occurring bilaterally, while 29 cases were described as occurring only on the anterior surface of the lower extremities (Table 4). Out of the 163 total cases of PP, 3.1% reported a single ulcerating lesion, 15.3% reported multiple ulcerating lesions, and 81% had no ulcerating lesions (Table 5).

**Table 4:** Distribution of PP PP: Pancreatic panniculitis

Location	Number of Lesions
Lower Extremity	152
Bilateral Lower Extremity	135
Only Anterior Lower Extremity	29
Feet	3
Thighs	18
Groin	1
Buttock	11
Abdomen	18
Chest	9
Back	11
Upper Extremities	32
Hands	4
Head	1
N/A	2
Pressure Points	1

**Table 5:** Ulceration in PP PP: Pancreatic panniculitis

Number of Ulceration(s) Reported	Total Cases (%)
No Ulceration	132 (81.0)
A Solitary Ulcer	5 (3.1)
>1 Ulceration	25 (15.3)
Inconclusive	1 (0.61)

Management

The most common treatment was the administration of systemic steroids, used in 47 cases (28.83%). Other treatments included conservative/supportive care, antibiotics, surgical procedures for pseudocysts and/or pancreatitis, endoscopic retrograde cholangiopancreatography (ERCP), magnetic resonance cholangiopancreatography (MRCP), and the use of chemotherapy and/or octreotide (Table 6). Among the cases treated surgically, the most common procedure was a pancreatectomy, followed by pancreatoduodenectomy (Whipple procedure), and stent placement. Of the associated tumors treated with chemotherapeutic agents, gemcitabine was the most used, followed by folfirinox and cisplatin. Approximately 18% of patients received only supportive care. For the few cases treated with immunomodulators, azathioprine and mycophenolate mofetil were the most frequently used. A full list of all procedures used in the management of PP can be found in Table 7-10.

**Table 6:** Management of PP PP: Pancreatic panniculitis; I&D: Incision and drainage; ERCP: Endoscopic retrograde cholangiopancreatography; MRCP: Magnetic resonance cholangiopancreatography; NSAIDs: Nonsteroidal anti-inflammatory drugs; PPI: Proton pump inhibitors

Management Type	Number of Cases Using Management Type	Percent of Cases
Systemic Steroids	47	28.83
Topical Steroids	6	3.68
Immunomodulators	6	3.68
Surgical (Excision/Debridement/I&D) of Skin	9	5.52
Surgical Resection of Cancer	18	11.04
Surgical Procedure Pseudocyst/Pancreatitis	20	12.27
Radiation Therapy for Malignancy	1	0.61
Cholecystectomy	6	3.68
ERCP/MRCP	13	7.98
Conservative/Supportive ONLY (No Antibiotics)	29	17.79
Hospice Care	5	3.07
Octreotide	13	7.98
Chemotherapy	17	10.43
Antimicrobials	27	16.56
Plasmapheresis	2	1.23
NSAIDs	6	3.68
Cholecalciferol Adjunct	1	0.61
PPI and Pancrelipase	1	0.61
Telaprevir/Hepatitis Drugs	1	0.61
Sandostatin, Ulinastatin	2	1.23
N/A or Refusal or Other	4	2.45

**Table 7:** Onset of Skin Disease Relative to Pancreatic Disease in Patients With PP Associated With Carcinomas PP: Pancreatic panniculitis

Etiology	Number of Total Cases of PP (%)	Number of PP Cases With Skin Lesions Occurring Before Pancreatic Symptoms (%)	Number of PP Cases With Skin Lesions Occurring After Pancreatic Symptoms (%)	Number of PP Cases With Skin Lesions Occurring Simultaneously With Pancreatic Symptoms (%)	Number of PP Cases N/A (%)
Pancreatic Adenocarcinoma	10 (6.13)	6 (60)	4 (40)	0 (0)	0 (0)
Pancreatic Acinar Cell Carcinoma	27 (16.6)	15 (55.6)	9 (33.3)	2 (7.41)	1 (3.70)
Neuroendocrine Tumor of Pancreas	6 (3.68)	3 (50)	3 (50)	0 (0)	0 (0)
Pancreatic Carcinoma, Unspecified/Other	14 (8.6)	11 (78.6)	3 (21.4)	0 (0)	0 (0)
Intrahepatic Cholangiocarcinoma	1 (0.61)	0 (0)	1 (100)	0 (0)	0 (0)

**Table 8:** Chemotherapy for Tumors Associated With PP PP: Pancreatic panniculitis

Chemotherapeutics Used	Number of Cases	Percent of Total Cases
Abraxane	1	0.61
Gemcitabine	7	4.29
Capecitabine	1	0.61
Cisplatin	3	1.84
Doxorubicin-Phosphate	1	0.61
Pazopanib	1	0.61
Larotrectinib	1	0.61
Oxaliplatin	2	1.23
Paclitaxel	1	0.61
Folfirinox	4	2.45
Nab-paclitaxel	1	0.61
5-FU	3	1.84
Irinotecan	1	0.61
Mitomycin C	1	0.61

**Table 9:** Immunomodulators Used for Treatment of PP PP: Pancreatic panniculitis; IVIG: Intravenous immunoglobulin

Immunomodulators Used	Number of Cases	Percent of Total Cases
Cyclophosphamide	1	0.61
Cyclosporine	2	1.23
Azathioprine	3	1.84
Hydroxychloroquine	1	0.61
Horse Anti-thymocyte Globulin	1	0.61
Tacrolimus	1	0.61
Mycophenolate	3	1.84
Rituximab	1	0.61
Thymoglobulin	2	1.23
OKT3 (Muromonab-CD3)	1	0.61
IVIG	1	0.61

**Table 10:** Procedures Used to Treat PP PP: Pancreatic panniculitis

Procedures	Number of Cases	Percent of Total Cases
Sphincterotomy	2	1.23
Whipple Procedure	8	4.91
Pancreatectomy	8	4.91
Amputation of Lower Extremity	1	0.61
Stent Placement	5	3.07
Frey's Procedure	1	0.61
Duct Dilation	3	1.84
Lavage	1	0.61
Laparotomy and Cyst Drainage	2	1.23
Surgical Bypass of Pseudocyst	1	0.61

Outcome

The most common outcome observed was the resolution of PP, which occurred in 63.8% of cases. Approximately 26% of cases of PP were associated with increased mortality (Table 11). In addition, 6% of cases showed partial resolution, 2% recurred, and an additional 2% had unknown outcomes (due to loss of follow-up) or other outcomes, such as amputation (Table 11).

**Table 11:** Outcome of PP PP: Pancreatic panniculitis

Outcome	Number of Total Cases	Percent of Total Cases
Complete Resolution	104	63.80
Death	42	25.77
Partial Resolution/Improvement	9	5.52
Recurrence	3	1.84
Unknown/Other	5	3.07
Total	163	100.00

Etiology and Lesion Ulceration

Pancreatic fistula was the most frequently identified underlying pathology in cases of PP with ulceration, occurring in 66.7% of cases (Table 1). Among cases with ulcerating lesions, neuroendocrine tumors of the pancreas were found in 50%, while pancreatic ACC was found in 29.6% (Table 1). Other etiologies associated with cutaneous ulceration include unspecified pancreatic carcinoma (21.4%), acute pancreatitis (19.1%), and unknown pancreatic disease (33.3%) (Table 1).

Timing of Skin Lesions and Pancreatic Disease Presentation

PP developed before, concurrently with, and after the underlying pancreatic disease in 44.8%, 5%, and 46.6% of cases, respectively (Table 12). Approximately 3.7% of reports did not describe latency (Table 12).

**Table 12:** Onset of Skin Disease Relative to Pancreatic Disease in Patients With PP PP: Pancreatic panniculitis

	PP Cases With Skin Manifestations Appearing Before Pancreatic Symptoms	PP Cases With Skin Manifestations Appearing After Pancreatic Symptoms	PP Cases With Skin Manifestations Appearing Simultaneously With Pancreatic Symptoms	PP Cases N/A
Number of Total Cases (%)	73 (44.8)	76 (46.6)	8 (4.9)	6 (3.7)

Timing of Lesions and Pancreatic Disease Based on Cancer Subtype

In 79% of carcinoma-associated cases, PP preceded the diagnosis of underlying malignancy. Specifically, in 60% of cases associated with pancreatic adenocarcinoma and in 55.5% of cases associated with pancreatic ACC, cutaneous findings preceded the diagnosis of the underlying tumor (Table 2). In contrast, patients with PP associated with neuroendocrine tumors of the pancreas were equally likely to develop skin findings either before or after the diagnosis of the underlying tumor (Table 2). A single case of intrahepatic cholangiocarcinoma was presented before the emergence of skin lesions (Table 2).

Etiology and Outcome

Half of all cases of PP associated with underlying pancreatic adenocarcinoma resulted in complete resolution, while 20% were associated with mortality. The most common outcome in cases associated with pancreatic ACC and neuroendocrine tumors of the pancreas was death, with mortality rates of 44.4% and 83.3%, respectively. About 64% of cases of PP associated with unspecified pancreatic carcinoma resulted in death. In contrast, 74.5% of cases with underlying acute pancreatitis resulted in complete resolution. Additionally, 91.7% of cases with unspecified pancreatitis and 87.5% of cases with chronic pancreatitis also resulted in complete resolution. Almost 90% of cases of PP due to pancreatitis secondary to other diseases resolved completely. Recurrence rates of PP were highest in cases associated with adenocarcinoma (10%) and pancreatic ACC (7.4%). A comprehensive review of each etiology and its associated outcomes can be found in Table 1.

Discussion

PP most frequently affects men in their sixth decade of life and typically presents on the lower extremities. While ulceration is considered a classic finding, we found that 81% of cases present without it [1]. The correlation between elevated pancreatic enzymes and the onset of PP symptoms underscores the role of pancreatic enzyme dysfunction in the pathogenesis of the condition. Recognizing the cutaneous manifestations as potential indicators of underlying pancreatic pathology is crucial, as these findings precede the diagnosis of pancreatic disease in 46.6% of cases.

The timing of the onset of PP is critical. In only 5% of cases, PP appears concurrently with pancreatic disease, while 44.8% of cases develop prior to the diagnosis of the underlying disease. We found that in over 50% of PP cases associated with pancreatic ACC, PP manifested prior to the underlying diagnosis of malignancy. Moreover, previous research by Rongioletti and Caputo has shown that PP is present in 16% of all pancreatic ACC cases, regardless of the timing of its onset [1]. These findings reinforce the association between PP and pancreatic ACC, highlighting the need for increased awareness when diagnosing patients with PP. Given the poor prognosis associated with pancreatic ACC, clinicians should consider PP as a potential early indicator of this malignancy and promptly investigate [7].

The findings in this review reaffirm that PP is a harbinger of a variety of underlying pancreatic pathologies. Notably, 30% of the PP cases were associated with acute pancreatitis, 16.5% were associated with pancreatic ACC, and 18.5% with hepatic metastases. Interestingly, the 28.8% of cases associated with acute pancreatitis in this study is significantly lower than the 54.2% reported by Betrains et al. in their recent systematic review [8]. Similarly, our findings are lower than the 49.6% of cases attributed to acute or chronic pancreatitis in a review by Zundler et al. [2].

It is important to note that Zundler et al.’s statistics included both acute and chronic pancreatitis, which may partially explain the elevated percentage of cases in their study [2]. However, even when we combined all cases of acute, unspecified, and chronic pancreatitis, the percentage of total cases associated with panniculitis in our review was still relatively low, at 41.1%. Betrains et al. also found that 11.9% of cases were caused by pancreatic malignancy, which is considerably lower than the 35.6% of cases found in this review [8]. This discrepancy may be partially explained by the differing inclusion criteria: Betrains et al. included only patients with a diagnosis of PPP syndrome, while our review did not require a diagnosis of polyarthritis for inclusion [8]. Despite these differences, both studies suggest that pancreatitis is the most common underlying cause of PP and PPP syndrome, regardless of the specific case percentage. Clinicians should be suspicious of pancreatitis in patients with PP and PPP syndrome. Clinicians should remain vigilant for underlying pancreatitis in patients with PP and PPP syndrome, but given the wide range of pathologies associated with PP, a full workup is essential.

The outcomes of PP are closely correlated with the underlying etiology. As expected, cases associated with acute pancreatitis had overwhelmingly positive outcomes, while those associated with neoplasms had much poorer prognoses. Only 19.15% of cases associated with acute pancreatitis resulted in death, while 74.47% resolved completely. Neuroendocrine tumors of the pancreas had the poorest prognosis among the neoplastic etiologies, with 83.33% (five of six cases) resulting in death. Other neoplastic etiologies showed somewhat similar outcomes: 44.44% of pancreatic ACC cases resulted in death, 64.29% of unspecified pancreatic carcinoma cases resulted in death, and 20% of pancreatic adenocarcinoma cases resulted in death.

In contrast, nearly 88% of PP cases attributed to chronic pancreatitis and 90% of cases linked to pancreatitis secondary to other diseases resulted in complete resolution. Overall, 63.8% of total cases were resolved completely, while 25.8% of the cases resulted in death. The percentage of cases that resolved completely is similar to the statistic reported by Betrains et al. (55.9% of cases after a median duration of six weeks) [8]. The mortality rate of 25.8% in our study is comparable to the 27.1% reported by Betrains et al. in 2021 and the 21.4% found by Zundler et al. in 2016 [2,8]. Since our study did not specifically include patients with polyarthritis, this may help explain some of the differences in mortality rates.

Limitations

A limitation of this article is the inconsistency in terminology and classification across the studies analyzed. The term "pancreatitis secondary to other diseases" was used in our statistical analysis to encompass smaller categories identified in these papers, such as "gallstone-related pancreatitis," "hepatic disease-related pancreatitis," "transplant pancreatitis," "azathioprine-induced pancreatitis," "Weber-Christian syndrome," and "autoimmune pancreatitis." Additionally, some studies lacked clarity in distinguishing between chronic and acute conditions, leading to our use of the term "unspecified."

## Conclusions

In conclusion, our review reinforces that PP is an important clinical sign that may precede the diagnosis of a variety of underlying pancreatic diseases, including both benign and malignant conditions. PP is often an early indicator of invasive pancreatic pathology, such as pancreatic adenocarcinoma and pancreatic ACC. Given that PP can manifest before the underlying pancreatic disease is diagnosed, clinicians must maintain a high level of suspicion when encountering unexplained skin lesions, particularly when associated with other risk factors for pancreatic disease, such as elevated pancreatic enzymes. Early recognition of PP offers a valuable opportunity for timely diagnostic workups, potentially leading to earlier detection and improved management of the underlying disease, especially when it involves aggressive malignancies like pancreatic ACC or other pancreatic cancers.

The management of PP requires a multidisciplinary approach, with treatment strategies focusing on both the skin manifestations and the underlying pancreatic pathology. Systemic steroids, surgical procedures, and targeted therapies based on the etiology of the pancreatic disease are commonly used. However, the resolution of PP is closely tied to effectively treating the primary pancreatic condition. Since PP can be associated with a broad spectrum of pancreatic pathologies, the collaboration between clinicians, dermatologists, and gastroenterologists is crucial for addressing the diagnostic and therapeutic complexities presented by this uncommon skin manifestation. Vigilant monitoring and individualized care are essential to optimize patient prognosis and prevent the progression of the underlying disease.
